# Key Molecular Requirements for Raft Formation in Lipid/Cholesterol Membranes

**DOI:** 10.1371/journal.pone.0087369

**Published:** 2014-02-03

**Authors:** Davit Hakobyan, Andreas Heuer

**Affiliations:** Theory of Complex Systems, University of Muenster, Muenster, Germany; University of Waterloo, Canada

## Abstract

The lipid mixture of DPPC (saturated lipid)/DUPC (unsaturated lipid)/CHOL (cholesterol) is studied with respect to its ability to form liquid-ordered and liquid-disordered phases. We employ coarse-grained simulations with MARTINI force field. All three components are systematically modified in order to explore the relevant molecular properties, leading to phase separation. Specifically, we show that the DPPC/DUPC/CHOL system unmixes due to enthalpic DPPC-DPPC and DPPC-CHOL interactions. The phase separation remains unchanged, except for the formation of a gel phase at long times after decreasing the conformational degrees of freedom of the unsaturated DUPC. In contrast, the phase separation can be suppressed by softening the DPPC chains. In an attempt to mimic the ordering and unmixing effect of CHOL the latter is replaced by a stiff and shortened DPPC-like lipid. One still observes phase separation, suggesting that it is mainly the rigid and planar structure of CHOL which is important for raft formation. Addition of an extra bead to the head of CHOL has no notable impact on the phase separation of the system, supporting the irrelevance of the Umbrella model for the phase separation. Reduction of the conformational entropy of CHOL by stiffening its last bead results in a significant increase of the order of the DPPC/CHOL domain. This suggests that the conformational entropy of CHOL is important to prohibit the gelation process. The interleaflet interactions as mediated by the terminal molecular groups seem to have a strong impact on the possibility of a subsequent gelation process after phase separation.

## Introduction

Among the variety of entities including lipids and proteins from which a complicated biological membrane is usually formed, cholesterol (CHOL) plays quite a special role in the creation of membrane rafts [1], [2]. Since biological membranes are complicated mixtures that are very difficult to analyze, many investigations are performed on model membranes containing either pure components or well-controlled mixtures of either two or three components [3]. Direct visualization of raft-like domains in model bilayer membranes has provided a tangible proof for the coexistence of liquid-ordered (Lo) and liquid-disordered (Ld) phases [4]–[6]. Of particular interest are synthetic membranes containing three components: saturated phospholipids, unsaturated phospholipids and cholesterol. In these model membranes one can observe raft like domains enhanced with cholesterol and saturated phospholipids.

Computer simulations of mixtures containing cholesterol and phospholipids employ different models that describe the molecular interactions on a different level of detail. With help of the well-established coarse-grained (CG) MARTINI potential [7], [8] the process of raft formation between DPPC (1,2-dipalmitoyl-sn-glycero-3-phospocholine), DUPC (1,2-dilinoleoyl-sn-glycero-3-phosphocholine) and CHOL could be demonstrated [9]. Recently, a stable phase separation for the DPPC/1,2-dioleoyl-sn-glycero-3-phosphocholine (DOPC)/CHOL MARTINI mixture was observed for relatively high DPPC and CHOL concentrations at 290 K temperature [10]. Some attempts to understand the driving force of phase separation as a rational between enthalpy and entropy of CHOL and phospholipids have already been undertaken. For example, Zhang et al. based on calculations of the energy suggested that the distinction of the phase and, particularly, condensed complex from non-condensed complex cannot be distinguish only by analysis of nearest neighbors [11]. Moreover, the free energy of CHOL transfer between palmytoyl-oleoyl-phosphatidylcholine (POPC) and sphingomyelin (SM) environments suggested that the aggregation of saturated lipids with CHOL molecules into the Lo phase raise from the influence of CHOL on lipid-lipid interaction and not by direct lipid-CHOL interaction [12]. Davis et al. very recently studied the DPPC/DUPC/CHOL system as well as related system with different unsaturated lipids [13]. Through systematic tweaking of the interactions between the hydrophobic groups of the lipids and CHOL molecules and studying the effect on the unmixing behavior they, particularly, concluded that the phase separation is driven primarily by the difference in interaction between the beads of saturated and unsaturated lipids as compared to the interaction of the beads between saturated lipids. In particular they showed that the DPPC/DUPC/CHOL system no longer phase separates after matching the interaction of the DPPC and the DUPC beads. Thus, the different conformational properties are not sufficient to drive the phase separation. The authors question the relevance of lipid conformational degree of freedom, to the phase separation which we, at least partially, try to answer in this paper. In the combined Monte-Carlo and Molecular Dynamics (MC/MD) work of de Joannis et al., the authors revealed a correlation between aggregation of DPPC with CHOL and the low tilt angles of CHOL [14]. A different facet of CHOL has been stressed by de Meyer et al. [15]. By using the method of dissipative particle dynamics the authors demonstrated that the phase separation is suppressed when an extra bead is added to the CHOL headgroup. This suggested that the Umbrella model, induced by the CHOL molecules on phospholipid headgroups [16] is of relevance for the raft formation. Understanding the relative impacts of different properties of lipids and CHOL on the phase separation is a challenging task that guided our analysis.

The goal of the present work is to study the relevance of the different enthalpic and entropic contributions as well as several structural properties of lipids and CHOL for the raft formation, using the MARTINI force field (FF). Here are some key questions, answered in this contribution: i) How do the conformational properties of the lipids change the nature of the phase separation? It turns out that the conformational properties of DPPC are of significant relevance whereas those of DUPC are unimportant. ii) What is the role of CHOL for the unmixing? Here we show that the key property of CHOL is its planar and rigid structure which can be emulated also by very different molecules. iii) When does the Lo phase end up in a gel phase? Here some specific properties of CHOL become relevant. In general, we aim to present a holistic view on the phenomenon of raft formation in the DPPC/DUPC/CHOL system.

Recently, via a detailed comparison of the MARTINI potential with a much more microscopic united-atom (UA) simulation we showed for the DPPC/DUPC/CHOL model system that the driving forces of the phase separation are very similar [17]. Perfect agreement of the MARTINI model with the UA system strongly suggests that the present results, obtained for the CG force field, also describe the key mechanisms of real systems.

## Materials and Methods

**Figure 1:**
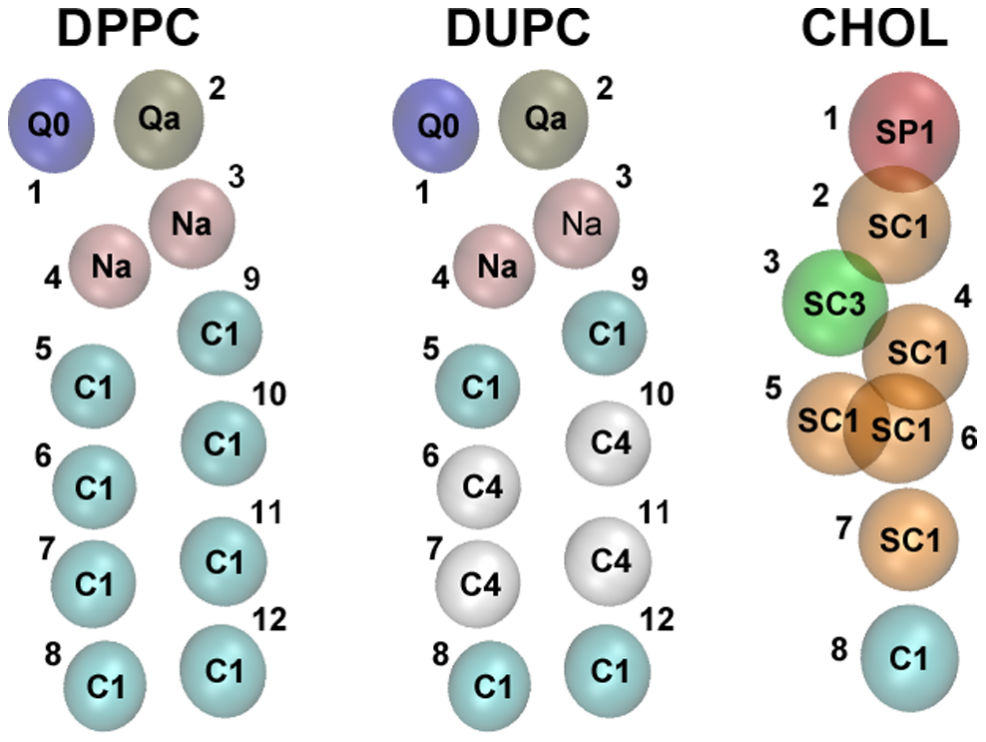
Martini CG models of DPPC, DUPC and CHOL. All the beads are colored according to their types and numerated. Types of all the beads are written within the beads.

For all the systems the concentrations 0.34∶0.51∶0.15 were used for DPPC/DUPC/CHOL or for their respective substituent. The CHOL (or its substituent) mole fraction of 0.15 was chosen similar to the system described elsewhere [17]. The MARTINI CG models of DPPC, DUPC and CHOL are presented in Figure 1. The beads are colored according to their types. The bead type C1 presents a single-bonded carbon group while C4 bead presents the double-bond region in the chain structure of DUPC. The SCx beads (where x can be any number from 1 to 5) present a ring structure in the CHOL molecule. For all the systems the bilayers were kept perpendicular toward the z direction during the simulations. The x/y side lengths of all the CG bilayers were ∼20 nm. In average, the systems were composed of 510 DPPC, 810 DUPC lipids, and 238 cholesterols or of their respective substituent. GROMACS version 4.5.1 was used for all simulations [18], [19]. The models of MARTINI potential 2.0 were used for lipids, cholesterol and standard water [8]. The systems were simulated by using an NPT ensemble with a semiisotropic pressure tensor of 1 bar. The Berendsen coupling scheme for the temperature and pressure was used [20]. Bond lengths were constrained by the linear constraint solver (LINCS) algorithm [21]. The systems were simulated at 295 K. The DPPC/DOPC +15% CHOL mixture was experimentally shown to form a liquid-liquid phases at temperature below 303 K [22]. On the other hand by interpolation of the experimental results the DPPC/DOPC +15% CHOL composition was suggested to separate into Lo/Ld phases at room temperature [23], [24]. Taking into account that in our systems the DUPC is used instead of the more ordered DOPC the range of temperatures of forming liquid-liquid phases should be even lower than that of the DOPC. As a result the simulated DPPC/DUPC/CHOL system at 295 K is well in the temperature range of Lo/Ld separation which has already been reported elsewhere [9], [17]. The initial random distribution of the original DPPC/DUPC/CHOL system was obtained by a 20 ns simulation at 450 K. This random configuration was used for all the other systems with modified DPPC or DUPC (by replacing the original component by its modified type). All the systems were simulated for 12 µs of effective times with a time step of 20 femtoseconds.

The shifted electrostatic and van der Waals (vdW) potentials were applied with 1.2 nm cut-off distance. Although the truncation of the long-range electrostatics in the atomistic level as opposed to the Particle-Mesh Ewald (PME) or reaction field techniques has been shown to cause artifacts [25], the CG methods bring the possibility for certain cases to adequately account the effect of long-range interactions in the short-range potentials [7], [8], [26], [27]. Despite the observation that the electrostatic interaction strength between the polar substances in the non-polarizable solvents is underestimated (due to the implicit screening with the MARTINI standard water model) [8], [28], nevertheless for simple bilayer system where no transition of a polar substance across an interface of different dielectric constants is studied the standard MARTINI water model demonstrates reasonable results [28]. Moreover, for MARTINI bilayer systems with standard water particles it was shown that using of PME for the long-range electrostatic interactions as compared with the cut-off scheme does not affect the lipid bilayer structure and dynamics [29] suggesting that the cut-off approach is a reasonable choice for the systems presented here.

In particular, to check the relevance of DUPC lipid to raft formation we study the impact of substituting DUPC by somewhat related molecules. All the modifications of the DUPC lipid targeted one or more of the three FF properties which differentiate DPPC from DUPC. Table 1 summarizes all the differences between DPPC and DUPC lipids. As shown in Figure 1 and listed in Table 1 the second and third beads in each chain of the DUPC structure (bead numbers 6, 7, 10 and 11) are more apolar (type C4) than the other beads of the chains and reflect the double-bond regions. This difference in bead types affect the enthalpic i.e. the vdW interactions between the DPPC and DUPC lipids. The modifications of DUPC are characterized by systematic assimilations of the angles, angle force constants (stiffnesses) and beads of the DUPC chains listed in Table 1 to the ones of the DPPC lipid. For the sake of clarity the DUPC modifications are named with respect to their differences from the DUPC. The lower letters ‘a’, ‘s’ and ‘b’ in the naming of the modified DUPC lipid stand for the angle, stiffness and bead, respectively. In the names of DUPC modifications which have bead type differences (i.e. DUb2, DUb3 and DUb23) the lower letter ‘b’ is followed by the number of the bead (or beads) in a chain which is different from the C4 type of the corresponding DUPC bead (or beads).

**Table 1:** Differences between DPPC and DUPC Lipids.

	DPPC		DUPC	
Differences	Bead number	Type/Value	Bead number	Type/Value
**Beads**	6–7, 10–11	C1	6–7, 10–11	C4
**Angles**	5–6–7 (9–10–11)	180°	5–6–7 (9–10–11)	100°
	6–7–8 (10–11–12)	180°	6–7–8 (10–11–12)	120°
**Angle force constants**	5–6–7 (9–10–11)	25 kJ/mol	5–6–7 (9–10–11)	10 kJ/mol
	6–7–8 (10–11–12)	25 kJ/mol	6–7–8 (10–11–12)	45 kJ/mol

Table 2 shows the corresponding vdW interaction strengths between the combinations of C1 and C4 pairs.

**Table 2:** vdW Interaction Strength (ε).

Bead type	C1	C4
**C1**	3.5 kJ/mol	3.1 kJ/mol
**C4**	3.1 kJ/mol	3.5 kJ/mol

The influence of chain stiffness of DPPC is also investigated. We consider a modified DPPC lipid for which the stiffness of the chain is decreased by 60% (10 kJ/mol instead of the default 25 kJ/mol). It is denoted DPPC_soft_. For the simulations with DPPC_soft_ the phase separated DPPC/DUPC/CHOL configuration was used as the initial structure of the new DPPC_soft_/DUPC/CHOL system.

It should be also noted that the interaction strength of SCx with type of Cy (where x and y may take any number from 1 to 5) is the same as the Cx-Cy interaction strength while the interaction of SCx-SCy is lower by a factor of 0.75 in respect to the strength of the corresponding Cx-Cy [8]. This aspect is used when discussing one of the substituents of CHOL (i.e. DPPC_3b_). The latter (DPPC_3b_) is introduced as shortened DPPC-like lipid. It has only three chain beads instead of the standard four and, most importantly, the angle force constants between the chain beads are set to a high enough value (here: 300 kJ/mol) to keep the chains straight. All the three beads of the chains were set to type SC1 to suppress the aggregation of DPPC_3b_ with itself as it is the case with CHOL. Additionally, an extra bond is added between the beads 7 and 11 to keep the chains together (see Figure 1 for the bead numbers).

The order parameter between A and B beads of the lipid chains is calculated as
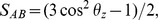



where θ_z_ is the angle between the AB bond and the z direction. The order parameter of a lipid is the average over all the consecutive beads in the chain of a lipid. For CHOL the θ_z_ is the angle between the line which passes through the CHOL beads 3 and 5 (Figure 1) and the z direction.

The domain correlation coefficients between bilayer leaflets are calculated by dividing the bilayer surface into square cells of side length 1.5 nm and calculating the correlation coefficients for the densities of the desired pairs in the corresponding cells of two leaflets.

The z coordinates of the head and tail beads of lipids and CHOL and their substituent relative to the bilayer normal were calculated by dividing the bilayer surface to square cells of side length 3.0 nm and calculating the bilayer center for each of these rectangular boxes by taking into account the z coordinates of only the tail beads of both lipids and CHOL and their “analogs” except for the DPPC_3b_. The latter is omitted from the calculation of the bilayer center since its shorted tail would result in shifting of the center position toward the higher concentration of DPPC_3b_ within each rectangular box.

The relevance of the Umbrella model for raft formation was elucidated by recording the CHOL molecules for which the head beads (bead 1 in Figure 1) were covered by the head beads of its nearest-neighbor lipids. To consider a lipid (or two lipids) covering the head of a CHOL molecule the head of the lipid had to be within the area of 0.25 nm side square above the CHOL head (or in case of two lipids, their heads had to be located within the area of 0.47 nm side square above the CHOL head).

The VMD package [30] was used for CHOL and lipids presentation.

## Results and Discussion

### General Properties of the Raft Formation

**Figure 2:**
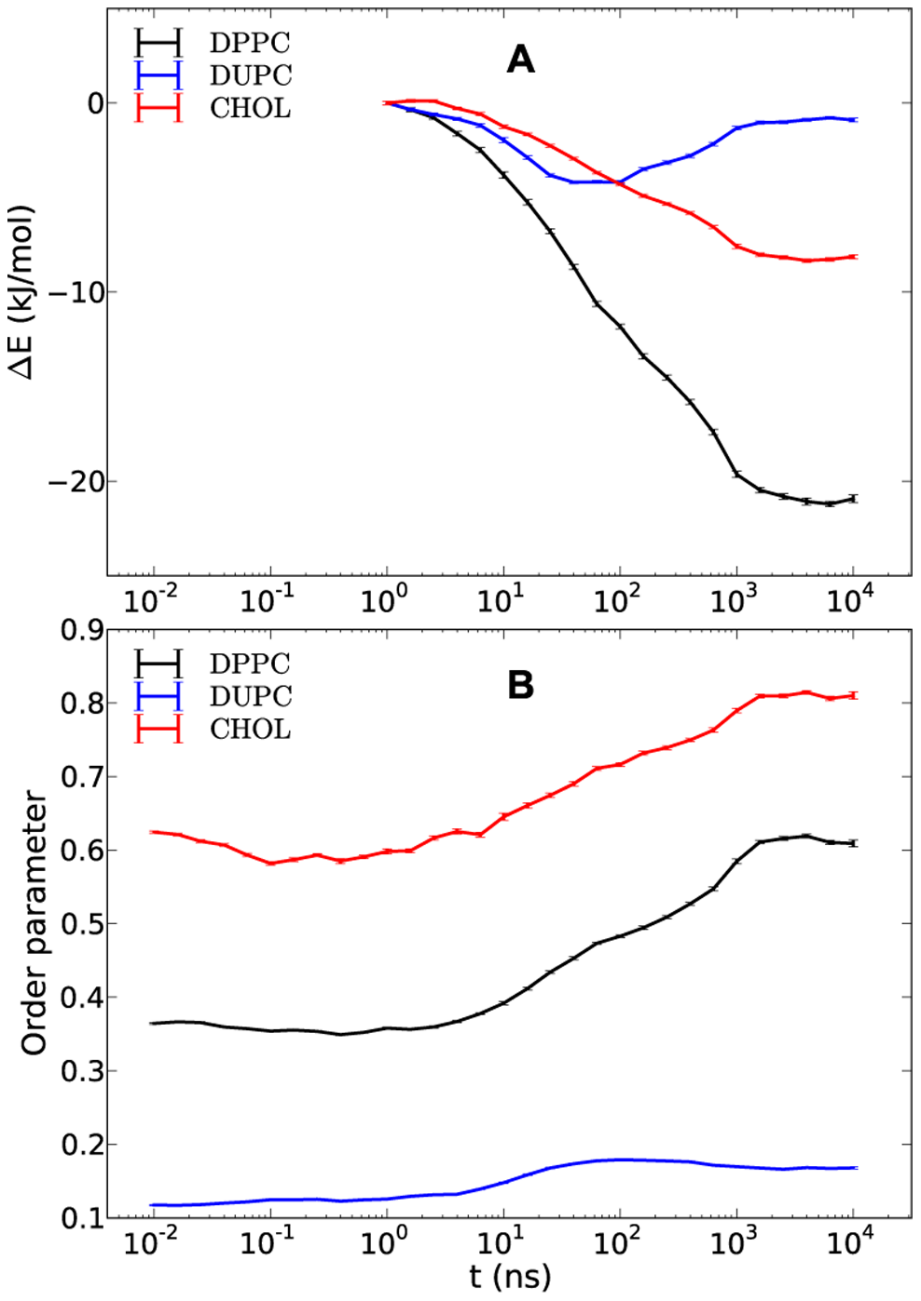
Time-dependent interaction energies and order parameters of DPPC, DUPC and CHOL. The interaction energy gains (A) present the differences between the absolute energies at a given time and the energies at time 1 ns. The average order parameters of DPPC, DUPC and CHOL are shown in (B). The small error bars present the standard errors around the values which are the averages of five independent runs.

We start by recording appropriate observables to describe the unmixing process somewhat closer. Figure 2 shows the total interaction energy variation of the DPPC, DUPC and CHOL as well as the respective order parameters. The energies are plotted relative to the time 1 ns because initial non-equilibrium effects, resulted from the preceding high temperature dynamics, have decayed. This has been verified by checking the absolute energy dynamics over time as well as calculating the root mean square deviation for the interval 0–10 ns taking as the reference structure the system state at 10 ns. The latter showed that after ∼1 ns the system is mostly equilibrated while the normal diffusion had not started yet. The energies include the pairwise vdW interaction and electrostatic contributions among the constituents of the bilayer and are normalized per molecule. The phase separation is reflected by a gain in enthalpy for DPPC (21 kJ/mol) and CHOL (8 kJ/mol) as well as by an increase of their order parameters. The latter might be considered as a qualitative characteristic of their loss of conformational entropy. In contrast, DUPC only shows very small variations with time. Our observations suggest that the unmixing behavior strongly depends on the properties of DPPC and CHOL and on the interplay between the enthalpy gain and the entropy loss. DUPC behaves very differently since the energy and the order parameter of the DUPC lipids are basically time-independent, except for the initial kink in the energy. In previous work it was already reported that also the diffusivity does not change during unmixing [17]. This might suggest that DUPC only plays a minor role for the mechanism of unmixing. However, as already reported by others the possibility of raft formation depends very sensitively on the enthalpic interaction of DUPC with DPPC [13]. Whether or not the conformational properties of DUPC chains play a crucial role will be also discussed below.

Naturally, in case of raft formation the free energy of the unmixed phase has to be lower than that of the mixed phase. Figure 2 suggests that the penalties of the entropy of mixing and the loss of conformational entropy of DPPC and CHOL (reflected by the increase of the order parameters) are overcompensated by the enthalpic gains of DPPC and CHOL. As already discussed above, DUPC hardly contributes to this comparison. Since the binary DPPC/DUPC system does not phase separate, the presence of CHOL has to play an important role in this free energy balance. One goal of this work is to obtain information, how this free energy balance is influenced by specific molecular properties.

### Relevance of DUPC

**Figure 3:**
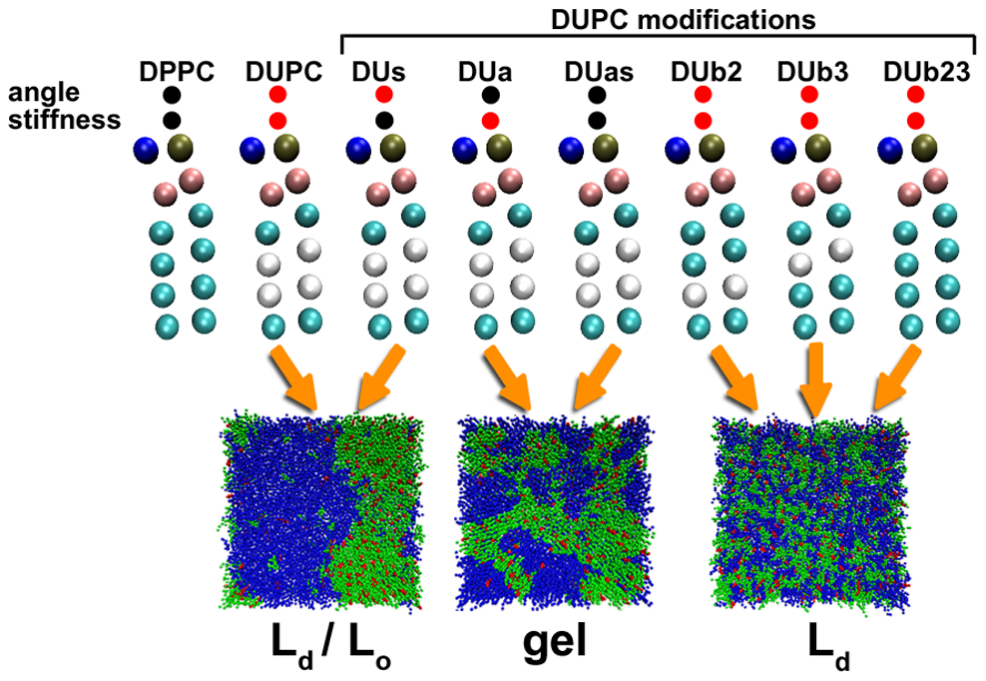
DPPC, DUPC and the modified DUPC lipids. The modifications relate to the equilibrium angle bonds and/or angle force constants (stiffness) as well as the bead types of the chains of DUPC. The types of the two middle beads of the lipid chains are set to C1 type (cyan) according to the corresponding DPPC bead type for the modified DUb2, DUb3 and DUb23 lipids. The black circles on top of the lipids indicate assimilations of the angles and/or angle force constants of the DUPC to the ones of the DPPC lipid while the red color indicates no change with respect to the original DUPC. The final configurations and phases of DPPC/DUPC/CHOL, DPPC/DUas/CHOL and DPPC/DUb23/CHOL systems after 12 µs of simulation time are shown at the bottom. These are characteristic configurations also for the other modified DUPC types as indicated by the arrows. In the snapshots, the DPPC is shown in green, the DUPC and its modifications in blue and the CHOL in red.

In the next step we start with the investigation of DUPC. Extending the work of Davis et al. [13] we not only vary the vdW interaction but also the angles of the lipid chains as well as their stiffnesses (Figure 3). In this way we systematically reduce the differences between DUPC and DPPC. Thus, replacement of the original DUPC by one of its modified lipids allows one to identify how a particular difference between DPPC and DUPC affects the final phase of the system.

The system with DUs lipids demonstrates only marginal differences as compared to the original system with DUPC lipids. Also the system with DUas as well as with DUa lipids displays phase separation. Rather than forming the Lo/Ld phase, a gel phase is formed in the course of the simulation (see also later discussion). Finally, the systems with modified bead types (DUb2, DUb3, DUb23) remain in the mixed Ld phase. The Dub23 behaves similar to the one of the DUPC modifications analyzed elsewhere [13] with only the difference of somewhat increased DUb23-DUb23 interaction as compared to DUPC-DUPC which, however, should not result in any notable changes.

As already noted by Davis et al. [13] the phase separation in the original DPPC/DUPC/CHOL system strongly depends on the DPPC/DUPC interaction. As soon as mixing is no longer energetically unfavorable for the vdW interaction, phase separation stops. Interestingly, changing the equilibrium angles or the angle stiffness of DUPC does not suppress the lipid unmixing which strongly indicates that the conformational degrees of freedom of DUPC are not important for lipid unmixing. This observation somewhat disagrees with the suggestion made by Lindblom et al. that the phase separation is guided by the increasing difficulty of unsaturated lipids with low order to be integrated in the DPPC environment of higher order [31]. Rather we observed that DUas despite its ability to fully order tends to separate from the DPPC/CHOL whereas DUb23 despite this conformational mismatch does not phase separate.

**Figure 4:**
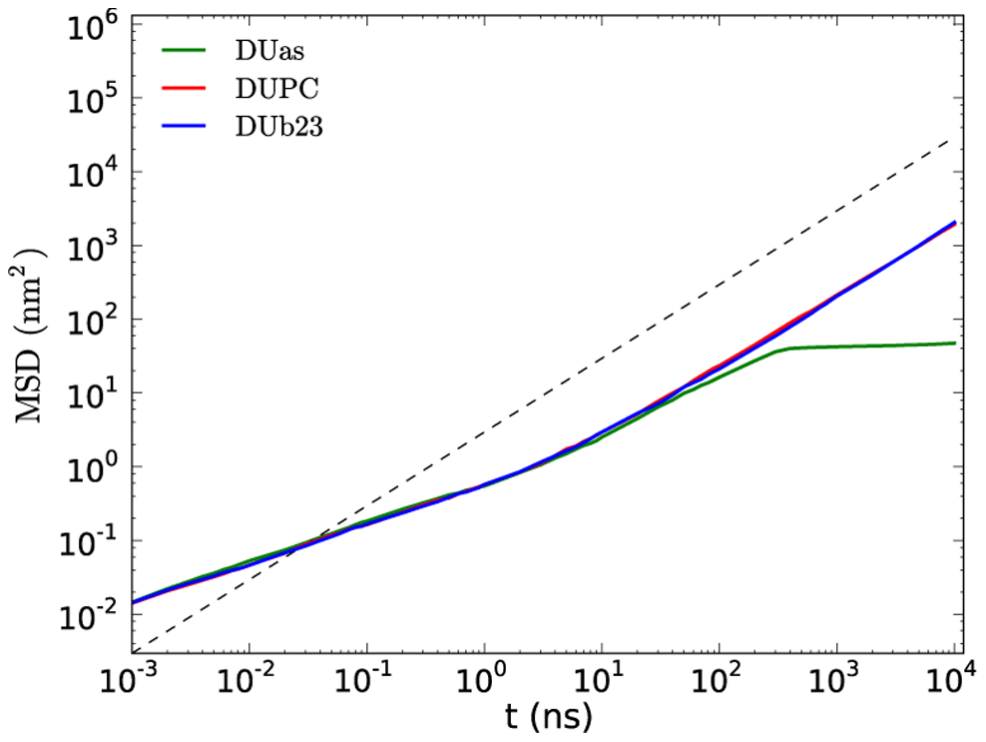
Time-dependent average MSD for DUPC, DUas and DUb23 over five independent runs. The dashed line has slope one, corresponding to pure diffusion.

Additional information about the time dependence can be extracted from the mean square displacement (MSD) of unsaturated lipids for the three representative systems (see Figure 4). Interestingly, up to the diffusive regime (∼20 ns) the MSD of all systems displays the same time-dependence. Thus, the diffusivity is neither influenced by microscopic details nor by the presence of phase separation. For longer times the MSD of DUas first starts to slow down and then (around 300 ns) remains constant, reflecting the immobile behavior of the gel phase. Thus, variation of the conformational degrees of freedom of DUPC is important for the behavior after the phase separation. Repeating this analysis for DPPC in the DPPC/DUPC/CHOL system the phase separation shows up as a significant reduction of the diffusivity, due to the lower mobility in the Lo phase [17].

**Figure 5:**
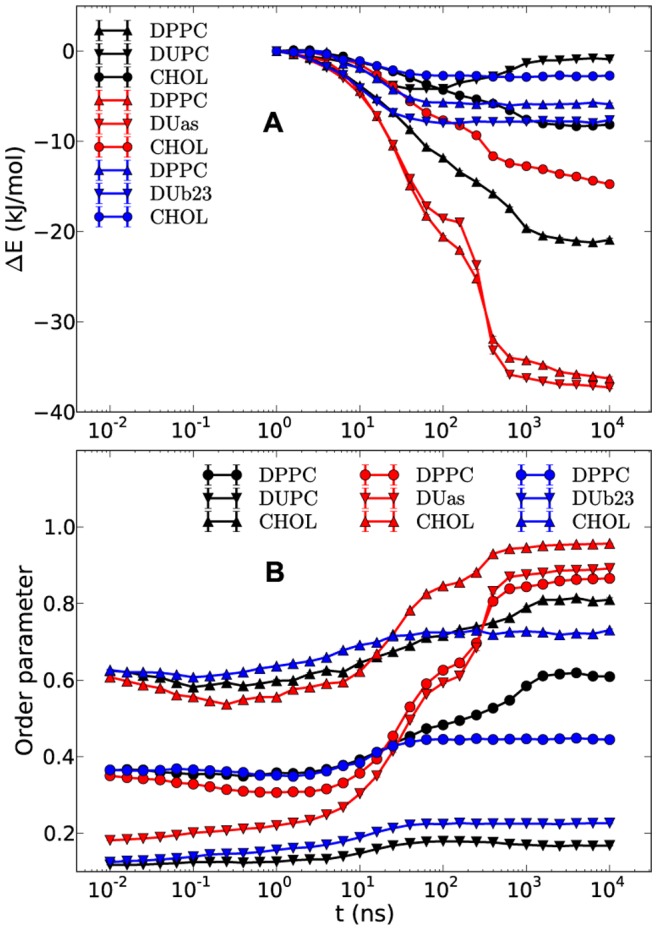
Time-dependent interaction energies and order parameters of DPPC/DUPC/CHOL, DPPC/DUas/CHOL and DPPC/DUb23/CHOL systems. The interaction energy gains (A) for each system present the differences between the absolute energies at a given time and the energies at time 1 ns. The average order parameters of the components for the three systems are shown in (B). The small error bars present the standard errors around the values which are the averages of five independent runs.

Figure 5 shows the time dependence of the individual energies and the order parameters for the three representative systems, namely DPPC/DUPC/CHOL, DPPC/DUas/CHOL and DPPC/DUb23/CHOL. As expected, each system displays a very different time dependence. The gel formation for the system with DUas shows up as a dramatic increase of the order parameters (around 0.9 for all three constituents), appearing as a two-step process. This high order parameter goes along with a dramatic decrease of the energy (−36 kJ/mol). The same observations (high order, low energy) are made for DPPC and CHOL. This is influenced by two factors, namely by the intraleaflet aggregation of DPPC and CHOL (or likewise of DUas lipids) and interleaflet interactions. While the latter contribution is discussed further below, for the former factor it should be noted that the order parameters of DPPC/CHOL and DUas composites during the phase separation is higher than the corresponding values of the DPPC/DUPC/CHOL system for two reasons. Firstly, the straight DUas chains induce less disorder over the surrounding DPPC and CHOL. Secondly, due to the same straight construct of DUas the vdW interaction between the DUas lipids should, in principle, be stronger than between the DUPC lipids which further speedup the unmixing.

In contrast, the DUb23 system only shows a mild increase of the order parameters. The interaction energy of DUb23 somewhat decreases with time and does not show the subsequent increase as observed for the DUPC system.

### Relevance of DPPC

So far we have seen that the enthalpic contribution, i.e. the detailed properties of the vdW interaction between DUPC and DPPC are relevant (as discussed elsewhere [13]) whereas the conformational properties of DUPC do not modify the tendency of phase separation. One might be tempted to conclude that, accordingly, the conformational properties of DPPC are irrelevant. This is not true as seen from the following results obtained for the DPPC_soft_/DUPC/CHOL system.

**Figure 6:**
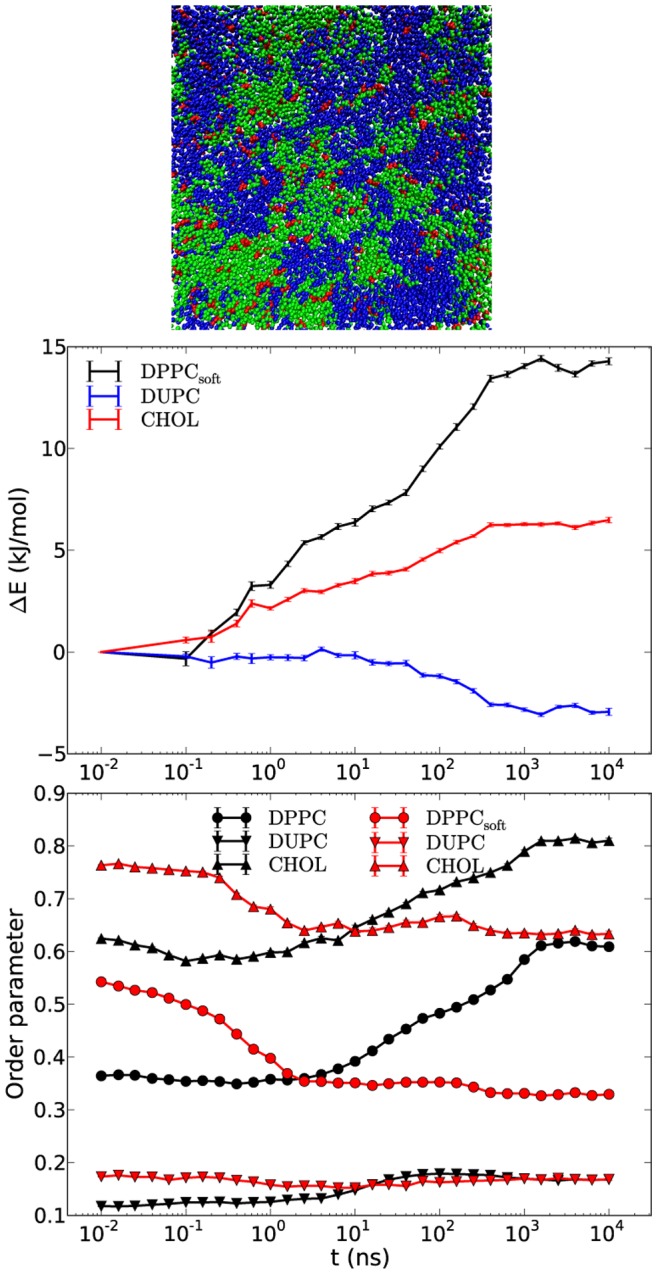
Snapshot, interaction energy gains and order parameters of DPPC_soft_/DUPC/CHOL system. The snapshot (top) presents one of the leaflets after 12 µs. The DPPC_soft_, DUPC and CHOL are colored in green, blue and red, respectively. The energies at time 0 are taken for references (middle). For the sake of readability the energy gain of 0 at time 0 is mapped to 10 ps. The order parameters (bottom) of the systems DPPC_soft_/DUPC/CHOL and DPPC/DUPC/CHOL are colored in red and black, respectively. The energy and order parameter data is averaged over three independent runs with standard error bars.

As shown in Figure 6 the phase-separated configuration starts to mix again when replacing DPPC by DPPC_soft_. More quantitatively, it turns out that the order parameters at late times are close to the values of the DPPC/DUPC/CHOL system before unmixing. This indicates that the Lo/Ld phases basically disappeared. Also the interaction energies display the opposite time evolution as compared to the DPPC/DUPC/CHOL system. The difference of the order parameters between the last points of CHOL and DPPC curves for the DPPC/DUPC/CHOL system and the respective first points of CHOL and DPPC_soft_ has a simple explanation. Due to the significant temporal equilibrium fluctuations of the order parameter, the order parameter of the selected configuration may somewhat deviate from the equilibrium value (e.g. 0.61 of the equilibrium value of DPPC at late times vs. 0.55 for the selected configuration for DPPC_soft_).

This result clearly shows that the conformational degrees of freedom of DPPC are of major importance for the raft formation. This can be easily rationalized. As mentioned above the free energy balance contains the gain in energy and the loss in entropy upon phase separation. A decrease of the force constants enables the exploration of a larger rotational phase space which, naturally, increases the conformational entropy of the disordered state. In contrast, the vdW interaction of DPPC chains in their ordered state is independent of these force constants. Thus, for the softer DPPC lipid the loss of entropy after ordering as compared to the gain in enthalpy becomes more unfavorable which effectively no longer supports phase separation from the thermodynamic perspective.

### Relevance of CHOL

Since the binary DPPC/DUPC system does not phase separate the presence of CHOL is indispensable. Various properties of CHOL like lipid ordering [32], [33], facial asymmetry [34]–[36], relatively frequent flip-flop across bilayer leaflets [37], [38], induction of the Umbrella effect of lipid headgroups [16], as well as the preference of CHOL to avoid direct interaction with itself [35], [38], have been implicitly or explicitly suggested to relate to the phase separation.

Obviously, two conditions are required that CHOL can trigger phase separation. First, the loss of conformational entropy upon entering the Lo phase has to be small. This is achieved by the rigid structure of CHOL. Second, the vdW interaction with surrounding saturated and ordered lipids has to be efficient in order to generate a significant enthalpy gain (overcompensating the loss of conformational entropy of the saturated lipid and the mixing entropy). For this aspect the relatively rigid and flat shape of CHOL is of utmost importance [30], [33], [39], [40].

It is worth noting that the partitioning of the CHOL enthalpy (Figure 2A) into pairwise interactions would show for total CHOL-CHOL interaction to be constant for the whole simulation run which just reflects the fact that CHOL is known to avoid direct interaction with itself [35], [38]. Thus, it is mostly the DPPC-CHOL interaction which leads to a decrease of the CHOL enthalpy. Zhang et al. suggested that association of CHOL with a saturated lipid such as sphingomyelin takes place because of the increasing gain of lipid-lipid interaction around CHOL [12]. The stronger decrease of DPPC enthalpy in Figure 2A also favors this suggestion. The enthalpic preference of CHOL to unsaturated lipid such as POPC rather than fully saturated lipid such as SM as further suggested by the same authors is likely not the case for polyunsaturated lipid like DUPC. This is implicitly supported by the observation that CHOL is found in the bilayer center in the polyunsaturated environment. [41], [42] For the MARTINI FF this is a direct consequence of vdW terms between the beads of CHOL and DPPC/DUPC (i.e. SC1-C1 and SC1-C4 interactions). Moreover, Davis et al. [13] showed that the increase of the vdW interaction terms of DUPC-CHOL up to the same level as for the DPPC-CHOL results in breaking of the phase separation. These observations indicate that not only the DPPC-DPPC interaction but also the direct DPPC-CHOL interaction takes part in the phase separation. The similarity of the driving forces of phase separations between the MARTINI and the UA models for the DPPC/DUPC/CHOL system [17] lets one suggest that this observation should likely be true not only for more detailed atomistic models but also for real systems.

**Figure 7:**
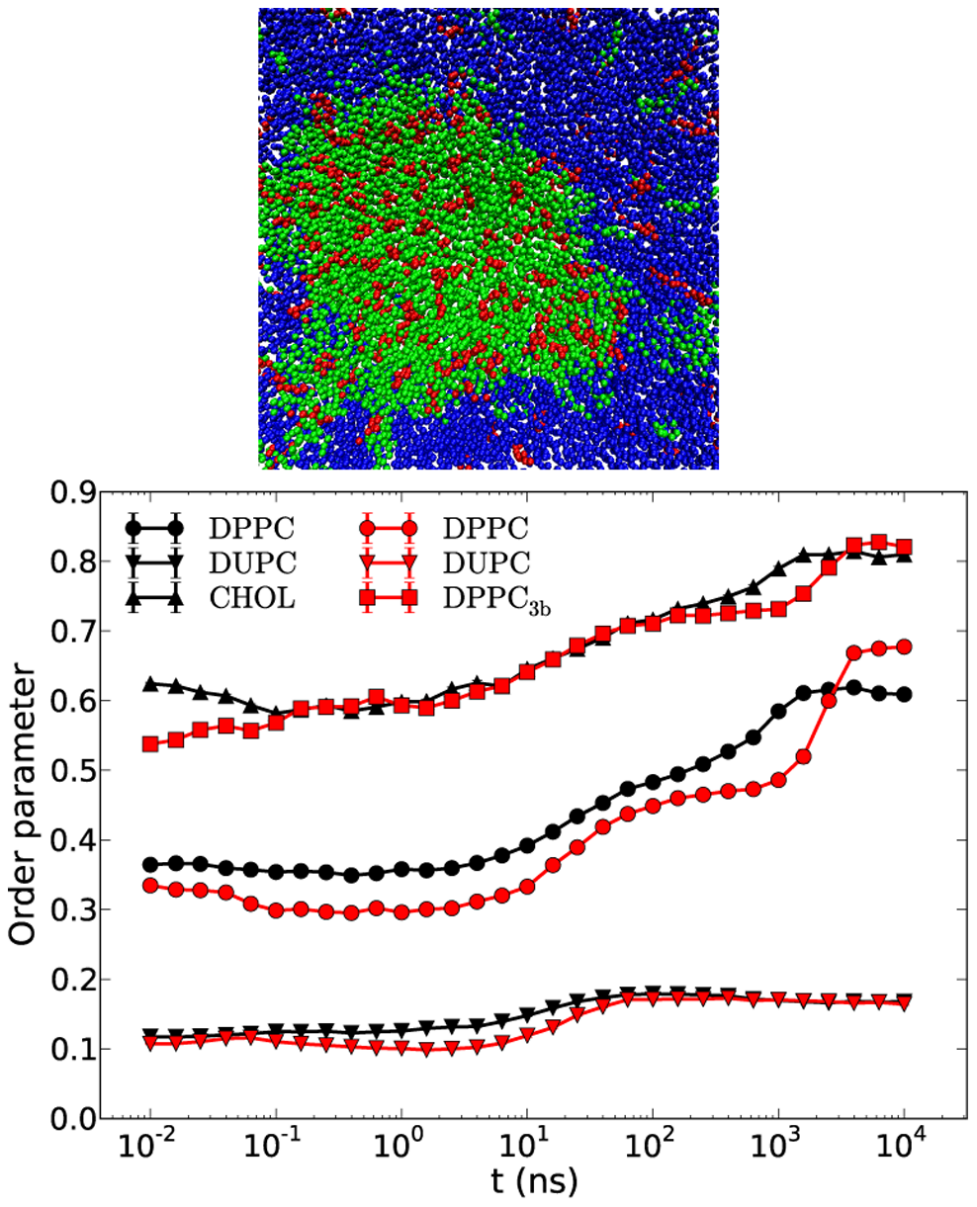
Snapshot and order parameters of DPPC/DUPC/DPPC_3b_ system. The snapshot (top) presents one of the leaflets after 12 µs. The DPPC, DUPC and DPPC_3b_ are colored in the snapshot in green, blue and red, respectively. The order parameters (bottom) of the systems DPPC/DUPC/DPPC_3b_ and DPPC/DUPC/CHOL are colored in red and black, respectively. The data of DPPC_3b_ system is averaged over three independent runs with standard error bars smaller than the marker symbols.

If the above arguments explain the relevance of CHOL one might think of alternative molecule which can serve the same purpose. Here, we mimic CHOL by replacing it by the DPPC_3b_ lipid analog. Figure 7 shows the snapshot of the new system and the order parameters for the DPPC/DUPC/DPPC_3b_ and DPPC/DUPC/CHOL systems. The system with DPPC_3b_ displays similar behavior as the original system in the sense of lipid unmixing and an increase in order. Thus, the key properties of CHOL can indeed be reproduced by the use of DPPC_3b_.

In some respect DPPC_3b_ is somewhat inferior to CHOL. First, for timescales less than 1 µs the ordering of DPPC lipids is weaker. Second, after unmixing, i.e. for time scales in the µs-regime, the DPPC order sharply increases, indicating the transition to the (sub-) gel phase (see next section for more details of the interleaflet interaction). Taking into account that the end beads of DPPC_3b_ chains are more distantly positioned from the bilayer center (by ∼0.4 nm) and that this distance increases beyond 0.6 nm with the ordering of DPPC chains it might be suggested that DPPC_3b_ brings an order to DPPC lipids similar to ordering of CHOL, but unlike the latter it brings no additional entropy to the system due to a lack of an additional mobile bead.

**Figure 8:**
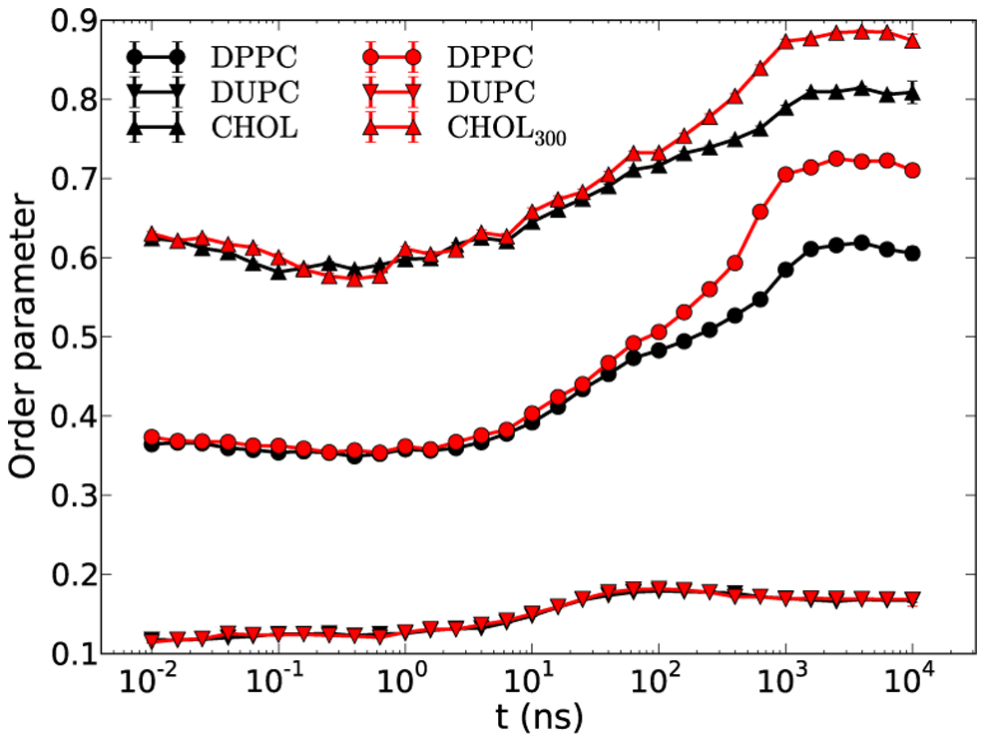
Order parameters of DPPC/DUPC/CHOL_300_ and DPPC/DUPC/CHOL systems. The order parameters of DPPC/DUPC/CHOL_300_ and DPPC/DUPC/CHOL systems are colored in red and black, respectively. The data of CHOL_300_ system is averaged over three independent runs with standard error bars smaller than the marker symbols.

According to these observations a supplementary simulation was conducted similar to the original DPPC/DUPC/CHOL system with the difference that the angle force constant of the last bead of the CHOL molecule (the angle is formed by the beads 4–7–8 of Figure 1) was increased from 25 kJ/mol to 300 kJ/mol (CHOL_300_). According to the above suggestion this stiffening might give raise to a further increase of the order parameter of DPPC. Indeed, as shown in Figure 8 for the DPPC/DUPC/CHOL_300_ system the order parameters of DPPC and CHOL_300_ show a rapid increase at long times which leads to (sub-) gel formation of the DPPC/CHOL_300_ domain. These results indicate that the Lo phase of the DPPC/CHOL domain is maintained not only by the rigid and planar structure of CHOL (due to which the order increases from Ld to Lo phase) but also is kept in the Lo phase due to a relatively mobile CHOL tail. Since this tail is heavily involved in the interleaflet interactions one may speculate that properties of the tail strongly influence the character of the interleaflet interaction. Whereas stiff end groups may support the interleaflet vdW interaction with the possible consequence of gel formation, more mobile end groups cannot overcome the entropic penalty of gel formation.

It is worth noting that addition of another bead to only one of the chains of DPPC_3b_ with angle force constant of 25 kJ/mol (similar to the angle force constant of 4–7–8 beads of CHOL) results in keeping the unmixed DPPC/DPPC_3b_ domain in the Lo phase which additionally supports our suggestion about the importance of the entropic contribution of the tail.

To emphasize the relevance of the discussed results of the MARTINI CHOL model to that of the atomistic (or real) CHOL molecule it is important to once again summarize the differences and the similarities of the MARTINI and the atomistic models in respect to the smoothness and asymmetry of the CHOL faces. As has already been discussed the direct CHOL-CHOL interaction for a MARTINI system is stronger than the one observed in an atomistic system [17]. Similar stronger direct interaction was observed between the demethylated (i.e. smooth and symmetric) atomistic CHOL (DCHOL) molecules [35], [36]. In this respect the MARTINI CHOL model assimilates to the smooth DCHOL. The ordering ability of the latter was shown to be inferior to that of the ordinary CHOL molecule. The degraded ordering of DCHOL (at moderate concentration) was related to the “linear” configuration which the DCHOL molecules form in a bilayer leaflet in contrast to the “triangular” configuration formed by the CHOL molecules. Remarkably, the MARTINI CHOL molecule despite its predominantly smooth faces was shown to also form a “triangular” configuration [17]. Thus, it can be concluded that the certain face asymmetry which is present for the MARTINI CHOL molecule [8] is enough for the formation of the “triangular” configuration which seems to be a higher order effect than the avoidance of the direct sterol-sterol interaction. The atomistic DCHOL molecule encompasses both of these two characteristics while the MARTINI CHOL model is somewhere between the CHOL and DCHOL. Nevertheless, the stronger CHOL-CHOL direct interaction for the MARTINI model as compared to the atomistic model has been advised to be insignificant in respect to the driving force of rafts [17].

### Role of Interleaflet Interaction

The MARTINI model has already been used to investigate gel formation in a pure DPPC bilayer [43] or in mixture of various lipids [10], [44]. The gel phase in those simulations where observed via nucleation or spinodal decomposition at different temperatures which were below the main phase transition temperature.

Here, for the systems such as DPPC/DUas/CHOL, DPPC/DUPC/DPPC_3b_ and DPPC/DUPC/CHOL_300_ we observed (sub-) gel phases for temperature of 295 K at which one should observe liquid-liquid phases for the DPPC/DUPC +15% CHOL composition.

Although, all the above three systems contained components which were in one or another way artificially stiffened, nevertheless, all they shared a characteristic dependence of gel phase upon interleaflet interaction. This was checked by simulating the asymmetric bilayers of these systems where one of the leaflets was completely occupied by DUPC lipids, thus strongly reducing the interleaflet interaction. In case of asymmetric DPPC/DUas/CHOL system the DPPC/DUas/CHOL layer still showed phase separation but ended up in a Lo/Ld phase rather than a gel phase. The final DUas order parameter was ∼0.35 which is larger than the original DUPC order parameter but, of course, much smaller than expected for a gel phase while the order of DPPC lipids aggregated with CHOL equilibrated at ∼0.5. Taking into account that in the asymmetric system the straight DUas lipids demonstrate about the same order at presence of higher ordered DPPC/CHOL domain as in case of pure DUas bilayer it might be suggested that DPPC interaction with DUas is rather insignificant. These observations suggest the highly non-trivial cooperative effect between DUas (DUPC) and DPPC/CHOL components and the relationship between intraleaflet aggregation and interleaflet interactions which result either in liquid-liquid phases or a gel phase.

**Figure 9:**
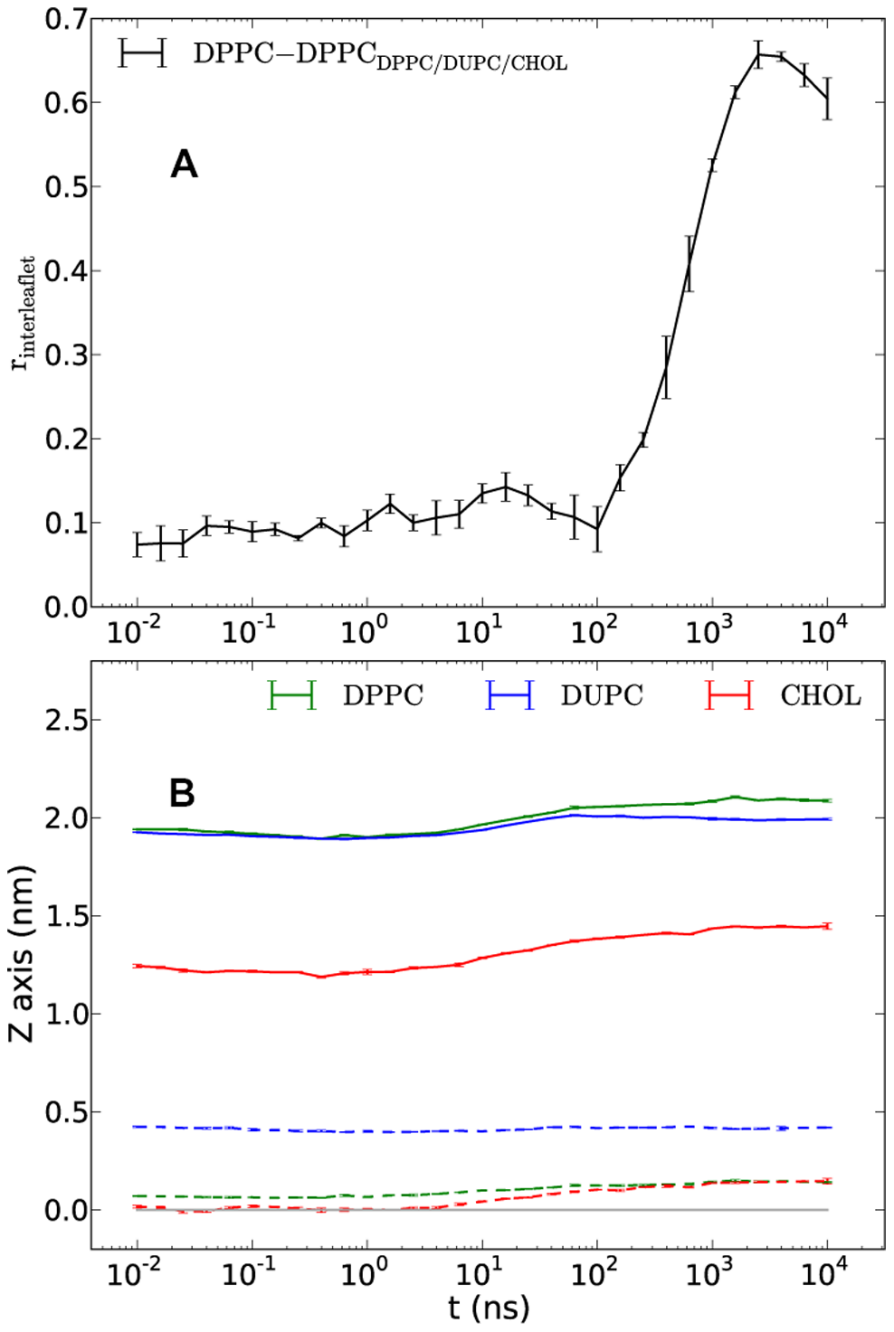
Interleaflet domain correlation coefficient and temporal z coordinates of lipid/CHOL heads and tails. Interleaflet DPPC-domain correlation coefficient (A) and temporal z coordinates of heads and tails of lipids and CHOL relative to the bilayer center for the DPPC/DUPC/CHOL system (B). The relative coordinates of heads and tails in (B) are shown in solid and dashed lines, respectively. The horizontal gray line at 0.0 shows the bilayer center. The error bars present the standard errors from averaging over five independent runs.

It is interesting to note that in contrast to the highly correlated positioning of the DPPC/CHOL domains across the leaflets of the original DPPC/DUPC/CHOL system shown in Figure 9A the DPPC/DUas/CHOL system demonstrates nearly no correlation having a value around 0.1 (data not shown). This could be expected since the correlation for the original DPPC/DUPC/CHOL system becomes notable only after a few hundred nanoseconds when significant aggregation of the DPPC.CHOL (or likewise of the DUPC) components had taken place (see Figure 5 of ref. 17). The same behavior applies to the DPPC/DUas/CHOL system where at the time of gelation, domains of considerable sizes in both leaflets started to show up (Figure 3). Keeping in mind that DUas are by construction as straight as DPPC and that the chain last beads of DPPC and DUas (DUPC) which participate in the interleaflet interaction are of the same C1 type the gel formation as a cooperative effect of intraleaflet aggregation and non-correlated interleaflet interactions becomes conceivable.

Another characteristic behavior of ordering is the tendency of the lipids/cholesterol tails in different leaflets to expand further away from each other. Figure 9B shows the average temporal positions of the lipids/cholesterol head and tail beads relative to the bilayer center for the DPPC/DUPC/CHOL system. The shown positions in z direction (bilayer normal) are the averages of two leaflets and by definition are symmetric across the bilayer center (the gray line). The last beads of DPPC and CHOL tails in both systems interdigitate, especially, in the initial - mixed state. On the first glance this could be surprising since interdigitation is supposed to introduce additional restriction to the bead movement thus favoring the chain ordering. On the other hand it is known that the lipid chain order decreases with approaching to the bilayer center. The chain tails being the most mobile chain parts bring both spatial restrictions as well as an additional entropy to the other leaflet during interdigitation, especially, when the latter is not very strong and involves mostly the last beads. Figure 9B basically show that the entropic influence of the tails in the x/y direction on the chains of the opposite leaflet is stronger than the spatial restriction effect. For the DPPC/DUas/CHOL system at late times the expansion from the bilayer center of the last beads of DPPC and CHOL is even stronger (data not shown) and reaches the value of ∼0.35 nm (the same for the tail end beads of DUas) thus becoming completely free of interdigitation. The separation distance of ∼0.7 nm between the last beads of the components in different leaflets together with the reduced mobility (as a result of certain ordering) introduce additional stabilizing vdW force on the tails of the lipid and CHOL molecules which eventually bring to gelation. The DPPC/DUPC/CHOL system after the phase separation still demonstrates small interdigitation (distance between the tail beads of different leaflets is ∼0.4 nm while the σ of the MARTINI vdW potential is 0.47 nm [7], [8]) which is in balance with the order of the system or rather with inability of the system to order further. It should be noted that the similar plot of head/tail positions for the united-atom (UA) DPPC/DUPC/CHOL system simulated previously for 9 µs and described elsewhere [17] shows no interdigitation of DPPC/CHOL tails in the mixed state. Their positions remains rather constant (only the positions of their head atoms i.e. nitrogen and oxygen atoms, respectively, slowly increase) at values of ∼0.24 and ∼0.2 nm for the DPPC and CHOL tail atoms, respectively (data not shown). This might be another reason of the large difference between the diffusivities of the CG and UA bilayers in the mixed states, which, however, is a subject of a separate study and is out of the scope of the present paper.

The correlation coefficient of positioning of DPPC and DPPC_3b_ against each other in different leaflets for the DPPC/DUPC/DPPC_3b_ system shows a considerable increase up to values ∼0.7 at late times (data not shown) which suggests that the sharp increase of the order parameter of DPPC lipid at presence of DPPC_3b_ is related i) to the absence of interdigitation of the end beads of DPPC_3b_ in contrast to the CHOL molecules (Figure 9B) and ii) additional stabilizing vdW force applies between the partially interdigitating end beads of DPPC and non-interdigitating end beads of DPPC_3b_.

The expansion distance of the end beads of DPPC and CHOL_300_ from the bilayer center at late times in the DPPC/DUPC/CHOL_300_ systems is very close to the corresponding distance for the original DPPC/DUPC/CHOL system. Thus, despite similar interdigitation the increase of the order parameters up to the (sub-) gel values (Figure 8) for the DPPC and CHOL_300_ is simply a consequence of the decrease of the entropy of the last bead of CHOL_300_ as compared to the ordinary CHOL molecules.

The separation of the asymmetric DPPC/DUPC/DPPC_3b_ and DPPC/DUPC/CHOL_300_ systems (where DUPC lipids fully occupy one of the leaflets) into Lo/Ld phases, once again, suggests the importance of the interleaflet interaction in formation of (sub-) gel phases.

**Figure 10:**
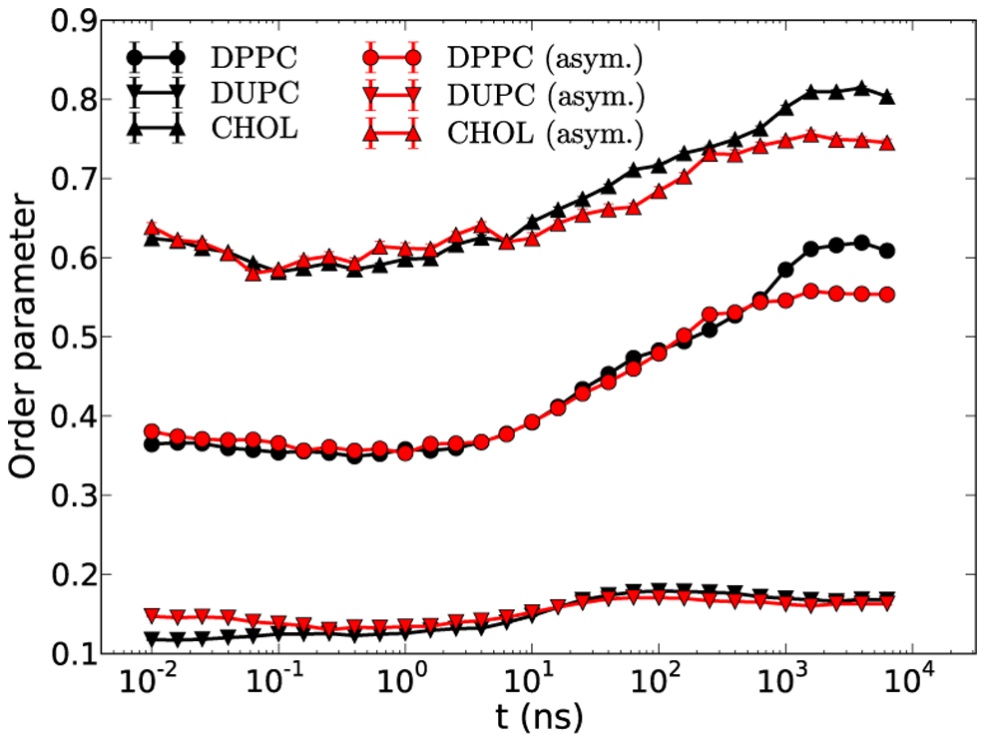
Order parameters of the symmetric and asymmetric cases of the DPPC/DUPC/CHOL system.

In case of the asymmetric DPPC/DUPC/CHOL system one still observes raft formation with slightly lower order parameter values as shown in Figure 10. Thus, the phase behavior in our standard system is not dominated by interleaflet interaction. The slightly higher order values at the late stage of unmixing for the symmetric DPPC/DUPC/CHOL system as compared to the asymmetric case is again a result of interleaflet interaction in the symmetric system and is similar to the behavior of the other systems with DUas, DPPC_3b_ and CHOL_300_ where such an increase of ordering is more pronounced.

**Figure 11:**
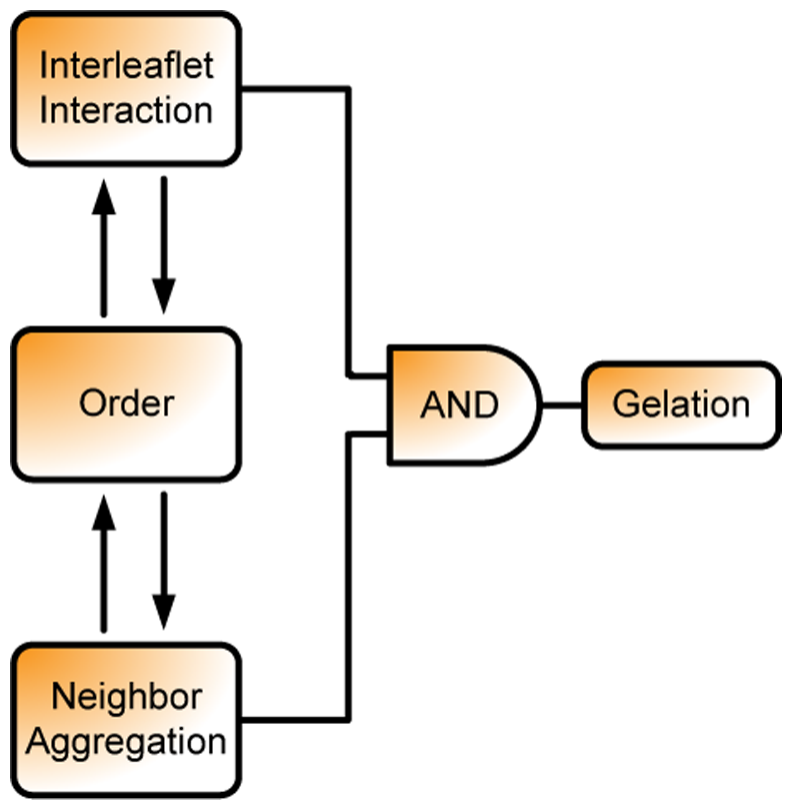
Diagram of the gel formation stresses the importance of interleaflet interaction.

The fact that the asymmetric systems do not end up in the gel phase indicates that the gelation occurs not due to solely nucleation of the stiffened components. To summarize, these examples suggest that the interleaflet interaction only mildly (if at all) determines the thermodynamic driving force for phase separation but may be relevant for the question whether the Lo phase may end up in a gel phase as sketched in Figure 11.

### Relevance of Umbrella Model

**Figure 12:**
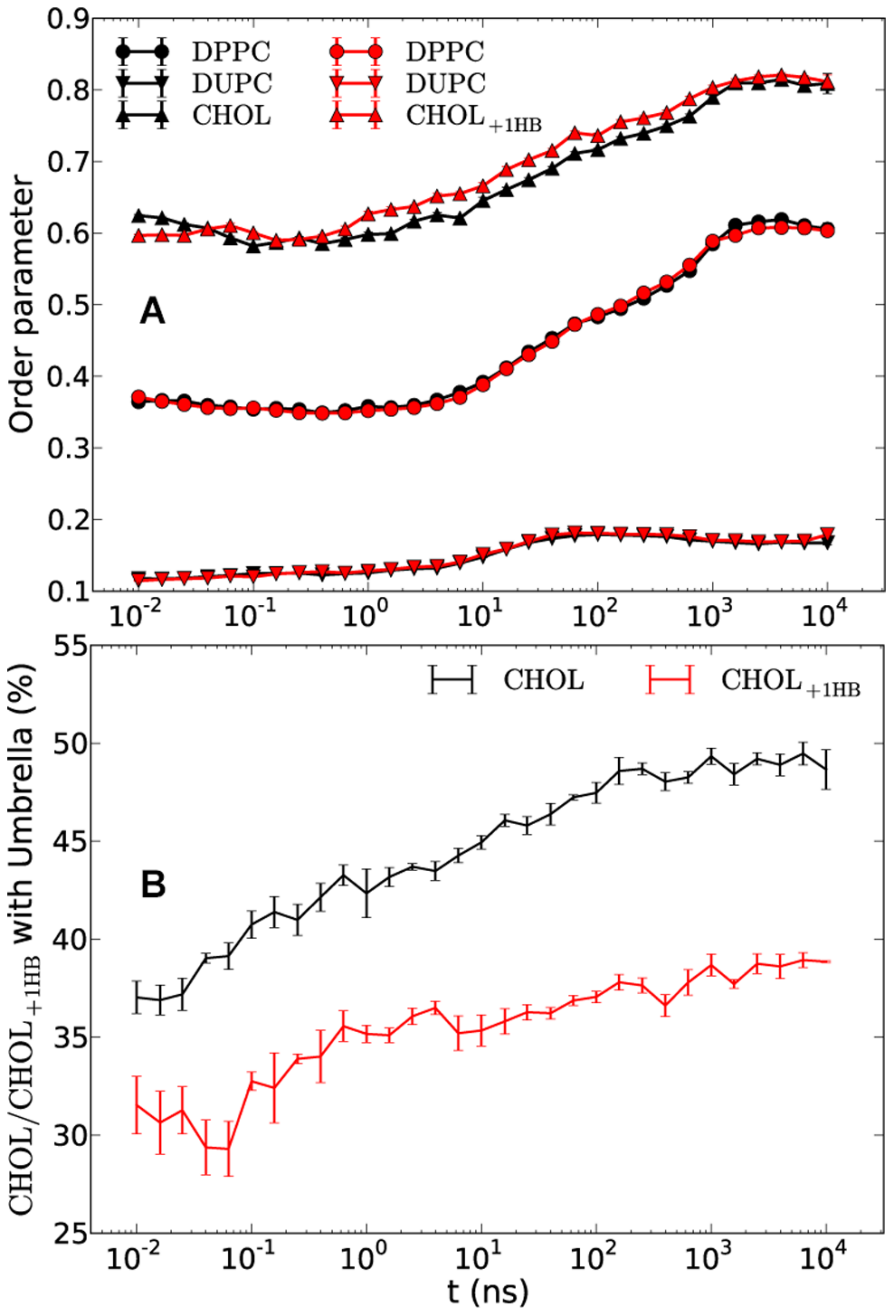
Order parameters and Umbrella model for CHOL and CHOL_+1HB_. The order parameters of DPPC/DUPC/CHOL_+1HB_ (red) and DPPC/DUPC/CHOL (black) systems (A) and the temporal ratios of all those CHOL or CHOL_+1HB_ molecules which are covered by one or two lipid heads i.e. belonging to the Umbrella model to the total number of sterols in the systems (B). The data is averaged over five and three independent runs for the systems with CHOL and CHOL_+1HB_, respectively The error bars show the standard error.

The condensing effect of CHOL has been suggested to favor from the coverage of CHOL by the polar headgroups of phospholipids [16]. With help of dissipative particle dynamics (DPD) it has been suggested that adding an extra bead to the head of CHOL results in breaking the formation of the Lo/Ld phases which was explained to be a result of breakage of Umbrella model and consequently a suppression of the “condensing” effect [15]. However, according to the results obtained for the DPPC/DUPC/DPPC_3b_ system, the DPPC ordering seems not to depend upon Umbrella model. To check this explicitly an extra polar bead (of type SP1) was added to CHOL (CHOL_+1HB_) and simulated in the same DPPC/DUPC mixture as the original DPPC/DUPC/CHOL system. As shown in Figure 12A the MARTINI DPPC/DUPC/CHOL_+1HB_ system displays nearly identical results as compared to the original CHOL. In Figure 12B the amounts of CHOL and CHOL_+1HB_ which can be considered influenced by the Umbrella effect are shown. One might notice considerable decrease of the Umbrella effect for the CHOL_+1HB_ as compared to the CHOL molecules in contrast to basically identical order parameters for the two systems. This additionally suggests that the ordering effect of CHOL on DPPC does not really depend on the ability of DPPC to cover the CHOL headgroup. Actually, this somewhat contradicts previous implications [15] but is concordant with the suggestion that the rigid and planar body of CHOL induces the order of DPPC.

The Umbrella effect was also calculated for the CHOL molecules of the UA DPPC/DUPC/CHOL system applying the same procedure (as described in the Methods) on the UA system reported earlier [17]. During the whole 9 µs simulation time only the half of the CHOL molecules appeared to be under lipid “umbrella” (data not shown) which agrees with the MARTINI CG model rather well taking into account the differences of the DUPC lipids between CG and UA models (the more disordered CG DUPC as compared to the UA DUPC) as was discussed in details elsewhere [17].

It would be interesting to separately study the influence of the polarized MARTINI water models on the bilayer “ability” to phase separate and, particularly, its impact on the tilt angle of CHOL and the Umbrella model.

## Conclusion

The DPPC/DUPC/CHOL mixture as well as carefully chosen variants of the three constituents was investigated via molecular dynamics simulations with the coarse grained MARTINI FF. One key goal was to understand how the enthalpic and entropic properties affect the phase behavior. Of course, for CG force fields some entropic aspects on the microscopic level are already included in the enthalpic terms. Nevertheless, it is still very instructive to characterize both contributions for this FF.

We have shown that the phase separation is a complex interplay of enthalpic and entropic contributions, the latter being reflected by variations of the order parameters. The following are the key ingredients as identified from our simulations: i) the enthalpic (but not the conformational) mismatch between saturated and unsaturated lipids, ii) the stiffness and planarity of CHOL to allow for significant vdW interaction with the saturated lipid, iii) a sufficiently stiff saturated lipid in order to limit the loss of conformational entropy upon ordering.

The phase separation in DPPC/DUPC/CHOL and DPPC/DAPC/CHOL systems is mainly driven by enthalpy in accordance with the results reported elsewhere [13]. We additionally show that also information about conformational aspects is important for a profound understanding of raft formation. Particularly it was shown that decreasing the conformational freedom of the chains of DUPC lipids does not change the process of phase separation but increases the tendency of gel formation. In contrast, when softening the DPPC chains no stable unmixing is possible.

Replacement of CHOL molecules by stiff and shortened DPPC_3b_ lipids resulted in a similar lipid unmixing with eventual higher order of DPPC. The similarity of lipid unmixing lets us suggest that the ordering property of CHOL as well as the induction of the phase separation relies on its rigid and planar structure. Furthermore, reduction of the conformational entropy of CHOL by increasing the angle force constant of its last bead results in an increasing order of the DPPC/CHOL domain. This suggests that the residual conformational entropy of CHOL is an important ingredient in prohibiting the DPPC/CHOL domain to reach the (sub-) gel phase. Finally it was shown that addition of an extra polar bead to the head of CHOL and thereby alteration of the Umbrella effect (covering the CHOL head by lipid headgroups) has no notable impact on the phase separation in contrast to previous report [15].

In summary, we attempted to understand and highlight those properties of saturated and unsaturated lipids and CHOL which have strong influence on the formation of Lo/Ld phases. The easiness which MARTINI FF provides in tweaking various FF parameters and structures opens a vast area for investigation and exploration of possible aspects which could influence the processes in membrane/protein systems. This might help to further understand the phase separation in more complex systems with transmembrane or soluble proteins as well as provide a quick check-up of those properties which might also be tweaked in atomistic presentations or even in experiments.
